# Goal Setting and Attainment in a Randomised Controlled Trial of Digital Health-Assisted Lifestyle Interventions in People with Kidney and Liver Disease

**DOI:** 10.3390/nu17071183

**Published:** 2025-03-28

**Authors:** Dev K. Jegatheesan, William F. Pinzon Perez, Riley C. C. Brown, Nicola W. Burton, Amandine Barnett, Lindsey Webb, Marguerite M. Conley, Hannah L. Mayr, Shelley E. Keating, Jaimon T. Kelly, Graeme A. Macdonald, Jeff S. Coombes, Ingrid J. Hickman, Nicole M. Isbel

**Affiliations:** 1 Faculty of Medicine, The University of Queensland, Brisbane, QLD 4072, Australia; 2 Department of Kidney and Transplant Services, Princess Alexandra Hospital, Brisbane, QLD 4102, Australia; 3 Queensland Cyber Infrastructure Foundation, The University of Queensland, Brisbane, QLD 4072, Australia; 4 School of Human Movement and Nutrition Sciences, The University of Queensland, Brisbane, QLD 4072, Australia; 5 Centre for Research on Exercise, Physical Activity & Health, The University of Queensland, Brisbane, QLD 4072, Australia; 6 RECOVER Injury Research Centre, Faculty of Health and Behavioural Sciences, The University of Queensland, Brisbane, QLD 4072, Australia; 7 School of Applied Psychology, Griffith University, Brisbane, QLD 4111, Australia; 8 Centre for Mental Health, Griffith University Mount Gravatt, Brisbane, QLD 4122, Australia; 9 Centre for Online Health, The University of Queensland, Brisbane, QLD 4072, Australia; 10 Centre for Health Services Research, The University of Queensland, Brisbane, QLD 4072, Australia; 11 Department of Nutrition and Dietetics, Princess Alexandra Hospital, Brisbane, QLD 4102, Australia; 12 Translational Research Institute, Brisbane, QLD 4102, Australia; 13 Department of Gastroenterology and Hepatology, Princess Alexandra Hospital, Brisbane, QLD 4102, Australia; 14 ULTRA Team, The University of Queensland Clinical Trials Capability, Herston, Brisbane, QLD 4072, Australia

**Keywords:** goal attainment scale, metabolic syndrome, diet, exercise, randomised controlled trial

## Abstract

Introduction: Goal setting is an effective strategy in altering fitness and dietary behaviours. The goal attainment scale (GAS) is a patient-reported outcome measure that can be used to quantify goal achievement. The GAS has not been extensively assessed in lifestyle intervention trials. This study aimed to describe the goal setting process and assess the impact of a digital exercise and diet service and self-efficacy on goal attainment in people with chronic disease and at increased cardiometabolic risk. Methods: This study presents a single-centre, 26-week, randomised controlled trial (RCT) comparing standard care to digital health technologies (text messages, nutrition/exercise app, video consultations with dietitian and/or exercise physiologist). The comparator group was offered dietitian review (per standard care), and both groups received a wearable activity monitor. Individualised goal setting was facilitated prior to randomisation. Goal importance, performance measures, and self-efficacy were determined by participants. Goal outcome and ‘Change in GAS’ scores, reflecting the difference between baseline and follow-up performance, were calculated using validated formulae. Results: Goal setting was completed and reviewed by 66 participants, with a median age of 51 years and 56% being male. The most common goals related to weight loss (46%), fitness (29%), and diet (15%). Most participants (62%) reported improvements in their goals, with most improvements in dietary (71%), fitness (52%), and weight loss (39%) goals. There was no significant difference in goal outcomes between intervention and comparator groups (*p* = 0.99). There was, however, a significant correlation between nutrition self-efficacy and dietary goal achievement (*p* = 0.04). Conclusions: The novelty and feasibility of goal setting and attainment were demonstrated in this RCT of lifestyle interventions in people with chronic disease. Though the intervention did not significantly improve goal attainment, most participants reported improvements in their lifestyle goals. There were greater improvements in dietary goals than in fitness or weight loss goals. Participant-led goal setting with GAS and participant self-efficacy has potentially important applications in future lifestyle modification research and clinical implementation endeavours.

## 1. Introduction

Goal setting and tracking have proven to be an effective strategy in altering fitness and dietary behaviours in adults [1,2]. It is therefore an important consideration as an implementation strategy for interventions that rely on lifestyle-related behaviour changes. As Cullen et al. [1] propose, there are four steps to successful goal setting: (1) recognising the need for change, (2) establishing a goal, (3) attempting the goal and self-monitoring, and (4) self-reward. The authors highlight that step two is more likely to be effective when goals are specific, quantifiable, and achievable, and step three is influenced by self-efficacy, strategy, resourcing, and barriers. Self-efficacy is a domain-specific cognitive variable that surmises an ‘individual’s belief in their ability to perform a task or reach a goal successfully’ [3]. The goal attainment scale (GAS) is a validated patient-reported outcome measure (PROM) that can be used to quantify achievement of individualised goals [4]. Though regularly used in clinical practice and frequently reported in randomised controlled trials (RCTs) [5], the GAS has not been extensively assessed in lifestyle intervention trials.

The metabolic syndrome (MetS) is broadly characterised by central obesity, impaired glycaemia, dyslipidaemia, and hypertension [6]. The mainstay of treatment of MetS is through aggressive lifestyle modification, focusing on weight reduction, healthy eating, and increasing physical activity [7]. In population-based studies, people with MetS have up to a 5.9-fold higher risk of developing chronic kidney disease (CKD) [8] and an 11.5-fold greater risk of having metabolic dysfunction-associated steatotic liver disease (MASLD) [9]. The prevalence of MetS and its components is also high in people with established CKD (54–66%) [10] and chronic liver disease (42–69%) [11].

Digital health interventions have been evaluated in people with MetS and chronic disease (including people with kidney and liver disease), with growing evidence supporting their effectiveness in modifying diet and physical activity behaviours in these cohorts [12,13,14]. From the patient perspective, digital health services that support diet and exercise have been reported as being acceptable, helpful, and safe [15]. The ‘Utilising technology for Diet and Exercise Change In complex chronic conditions across Diverse Environments (U-DECIDE)’ study was an RCT conducted to assess the feasibility of a patient-centred digital health exercise and diet service in people with kidney or liver disease who had at least one feature of MetS [16,17]. The objectives of the current study were to identify lifestyle goals, describe the goal setting process and assess the impact of the intervention and self-efficacy on goal attainment in participants of the U-DECIDE study.

## 2. Methods

**Figure 1:**
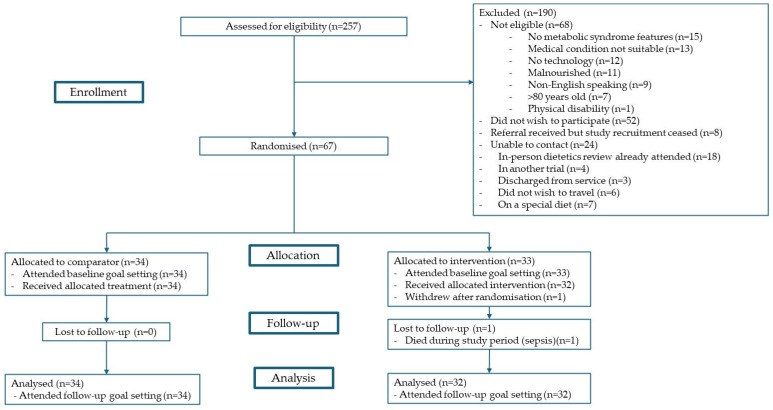
Participant flow.

The U-DECIDE study was a single-centre, 26-week, parallel RCT with a 1:1 allocation. The study was conducted between December 2020 and June 2022. Target participants were adults with kidney (CKD, haemodialysis, peritoneal dialysis, or transplant) or liver (chronic liver disease or transplant) disease who were at increased cardiometabolic risk (having ≥ 1 feature of MetS per the harmonised criteria [6]) and receiving specialist care at the Princess Alexandra Hospital in Brisbane, Australia. All participants received usual medical care and, additionally, were offered an initial dietitian consultation and a wearable activity monitor. The intervention group was offered access to a suite of digital health technologies, including text messages, a nutrition app, an exercise app, and video consultations with a dietitian and/or video consultations with an exercise physiologist. The comparator group was offered an individualised review with a dietitian (per standard care) with follow-up frequency as deemed clinically appropriate. These participants did not have access to the digital health technologies or exercise physiologists via the study. Further details on study design, eligibility criteria, recruitment, randomisation methods, and procedures of the U-DECIDE study have been outlined in detail in the protocol publication [16], and participant flow has been summarised in Figure 1. Of note, this study was conducted during the COVID-19 pandemic, which did have an impact on the rate and number of participants recruited. This study is reported in line with the CONSORT extension to pilot and feasibility trials [18] and Template for Intervention Description and Replication (TIDieR) [19] guidelines.

Goal setting was undertaken with all participants at the baseline visit, prior to randomisation. Participants were asked to generate up to two lifestyle goals that they hoped to achieve during the study. Investigator D.K.J., who was blinded to the group allocation, facilitated the goal setting, ensuring that the goals set by the participants honoured the SMART (Specific, Measurable, Achievable, Realistic, and Timely) principles [20]. The goal setting sessions were undertaken face-to-face, one-on-one (unless participants requested a caregiver to be present), in a private clinic room, and they lasted between 15 and 20 min per participant. The goal setting sheet was completed for each goal. Participants rated the ‘importance’ (‘A little important’ = 1; ‘Moderately important’ = 2; ‘Very important’ = 3) and ‘baseline performance’ score (perceived performance level at baseline; −1 or −2). All participants were given a copy of their goals and encouraged to place these in an area where they could regularly review the document (e.g., on the home refrigerator door, in a workspace). After the baseline visit, a ‘degree of difficulty’ score (‘A little difficult’ = 1; ‘Moderately difficult’ = 2; ‘Very difficult’ = 3) for each goal was determined by consensus by three members of the multidisciplinary investigator team (D.K.J., R.C.C.B., L.W., I.J.H., S.E.K., M.C.C., H.L.M.), including a physician, exercise physiologist, and dietitian.

The ‘follow-up performance’ score (‘Much less than expected’ = −2; ‘Less than expected’ = −1; ‘As expected’ = 0; ‘Better than expected’ = 1; ‘Much better than expected’ = 2) was determined for each goal by the participants at the 26-week post-study assessment. These sessions were facilitated by investigator D.K.J. and undertaken face-to-face, one-on-one (unless participants requested a caregiver to be present), in a private clinic room, and they lasted between 20 and 25 min per participant.

**Figure 2:**
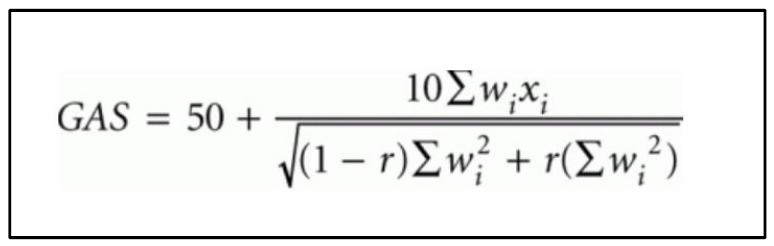
Goal attainment scale (GAS) formula as proposed by Kiresuk and Sherman [4]. wi = weight score assigned to the goal. Weight scores are added together if more than 1 goal. xi = performance score achieved (i.e., between −2 and +2). r = expected correlation of the goal scales, approximating to 0.3 [4].

Goal outcomes were defined as ‘Improved’ if follow-up performance—baseline performance score ≥ 1; ‘Same’ if follow-up performance—baseline performance score = 0; and ‘Regressed’ if follow-up performance—baseline performance ≤ −1. The more nuanced GAS scores (‘Baseline GAS’, ‘Outcome GAS’, and ‘Change in GAS’) consider the ‘Importance’ and ‘Degree of difficulty’ of each goal and were calculated and tabulated in an automated Microsoft Excel^®^ sheet, using the formula proposed by Kiresuk and Sherman (Figure 2) [4]. The ‘Weight’ score was calculated by multiplying the ‘Importance’ and ‘Degree of difficulty’ scores. A positive ‘Change in GAS’ score indicated an improvement in performance; a negative score indicated worsening in performance; and a score of 0 indicated no change in performance on the goal(s) over the study period.

Self-efficacy was assessed at the baseline visit, using the Nutrition Self-efficacy Scale and the Physical Exercise Self-efficacy Scale [21]. The questionnaires were completed in person by all participants with assistance from investigator D.K.J. as required. The questionnaire responses were tallied to generate self-efficacy scores for nutrition and physical exercise for each participant (each out of a possible total of 20).

All goal setting and self-efficacy data were de-identified and transposed into the REDCap research management system [22] by the research project officer (L.W.).

### Statistical Methods

Summary statistics were reported according to the nature of the variable and its distribution. For continuous variables, the following statistics were reported: number of observations (N), mean, standard deviation (SD), median, interquartile range (IQR), and range. Additionally, the distribution of each variable was tested using the Shapiro–Wilk test. If the variable was found to be normally distributed, the mean and SD were reported; otherwise, the median and IQR were reported. For categorical variables, the counts for each category of the variable were presented, as well as their corresponding percentages. The relationship between the change in GAS scores and the nutrition and physical exercise self-efficacy scores was explored using correlation analysis, with the Pearson’s correlation coefficient being estimated if the variables followed a normal distribution or Spearman’s correlation coefficient otherwise. The relationship between the continuous variables of interest baseline GAS score, outcome GAS score, change in GAS score, nutrition self-efficacy, physical exercise self-efficacy, and the total number of goals by goal type; the categorical variables treatment group, gender, and chronic disease were estimated in pairs with the non-parametric Mann–Whitney U test since all of the observed variables were not normally distributed. Fisher’s exact test was performed to compare goal categories, and the Chi-squared test was performed to compare goal improvements between treatment groups. *p*-values as well as measurements of effect size (Cohen’s W for Chi-squared test, Cohen’s D for Mann–Whitney test) and the statistical power achieved by the analyses were presented. All data analyses were performed in R version 4.3.3 with RStudio Desktop version 2023.6.0.421 (R Foundation for Statistical Programming, Vienna, Austria). Statistical significance was set at *p* < 0.05.

## 3. Results

Baseline demographic and medical details of the participants are summarised in Table 1.

### 3.1. Goal Setting and Achievement

**Figure 3:**
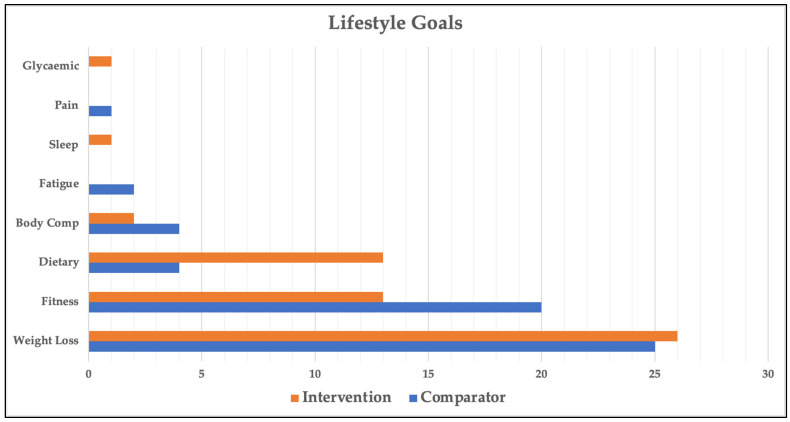
Lifestyle goal categories across groups. Glycaemic—improve glycaemic control; Pain—reduce pain; Sleep—improve sleep; Fatigue—reduce fatigue; Body Comp—body composition.

Goal setting was completed by all 67 participants; however, 1 participant died prior to the 26-week post-study goal review session and was excluded from all goal setting and goal attainment analyses. Of the 66 participants analysed, 46 chose 2 goals and 20 chose 1 goal (112 goals in total). The categories and distribution of participant-generated goals are shown in Figure 3. The most common goal across all participants was related to weight loss (46% of goals). The next two most common goals were related to fitness (29%) and diet (15%). There was a statistically significant difference in goal categories between the intervention and comparator groups (*p* = 0.04, Cohen’s W = 0.34, power = 74.8%), most notably in dietary (7% vs. 23%) and fitness (34% vs. 27%) goals. Table 2 provides more granular detail on participants’ dietary and fitness goals.

Most participants (62%) reported improvement in their overall goal outcomes (sum of goal 1 and goal 2 [if applicable]) over the study period, with no significant difference between the intervention and comparator groups (20/32 in intervention group and 21/34 in comparator group; *p* = 0.99, Cohen’s W = 0.008, power = 5.04%). Of the 112 individual goals that were set across all participants, 55 (49%) were reported as improved, 49 (44%) remained the same, and 8 (7%) regressed over the study period. Dietary goals were the most improved, with 12/17 goals (71%) reported by participants as improved over the study period. In contrast, a lower proportion of goals relating to fitness (17/33 [52%]) and weight loss (20/51 [39%]) were reported as improved. This was inversely proportional to the degree of difficulty of the goals, with the average degree of difficulty of 1.59, 1.92, and 2.33 (out of 3) for dietary, fitness, and weight loss goals, respectively.

### 3.2. Goal Attainment Scale Scores

There were no significant differences between the intervention and comparator groups for baseline GAS (*p* = 0.54, Cohen’s D = 0.22, power = 14.1%), outcome GAS (*p* = 0.71, Cohen’s D = 0.18, power = 10.8%), or change in GAS (*p* = 0.58, Cohen’s D = 0.25, power = 16.9%) scores (Table 3).

### 3.3. Self-Efficacy Scores

There were no significant differences at baseline between intervention and comparator groups for Nutrition Self-efficacy (*p* = 0.31, Cohen’s D = 0.32, power = 24.4%), Physical Exercise Self-efficacy (*p* = 0.28, Cohen’s D = 0.30, power = 21.6%), or Composite Self-efficacy scores (*p* = 0.85, Cohen’s D = 0.05, power = 5.4%) (Table 4). There was no significant correlation between baseline Physical Exercise Self-efficacy and change in GAS scores for those with fitness goals (correlation coefficient −0.12, *p* = 0.50, power = 8.3%). However, there was a statistically significant correlation between baseline Nutrition Self-efficacy and change in GAS scores for those participants who had dietary goals (correlation coefficient 0.51, *p* = 0.04, power = 87.9%) (Table 5).

## 4. Discussion

This study described the goal setting process and assessed the impact of a lifestyle intervention and participant self-efficacy on goal attainment. In this study, 62% of all participants with kidney or liver disease and with or at risk of MetS reported improvements in their lifestyle goals. Weight loss, fitness, and dietary goals were the most chosen, and the digital health diet and exercise services (intervention) did not change the ability for participants to attain their goals compared to usual care with their health practitioners. Compared with fitness or weight loss goals, participants more commonly reported improvements in dietary goals, which were also deemed less difficult to achieve. Higher nutrition self-efficacy scores at baseline were associated with improvements in dietary goals over the study period.

Goal setting, participant self-efficacy, and goal attainment have potentially important implications for future clinical and research endeavours, especially in the context of lifestyle modification. With almost two-thirds of participants improving in their lifestyle goals, and no significant difference between the comparator and intervention groups, the act of goal setting may itself have been a positive intervention, thereby improving motivation and accountability [23]. The SMART goal approach enables participants and facilitators to generate goals that are patient-centred and thus may ultimately be more likely to be achieved [24,25,26]. This would seem an important and necessary implementation strategy for any future clinical or research intervention involving lifestyle modification. An improved understanding of a patient’s specific goals may influence clinical decision-making and the way clinicians utilise digital health. For example, digital health was found to be effective in providing nutritional education, monitoring outcomes, and delivering interventions in a scoping review of nutritional management in people with cognitive impairment [27]. Goal categories, goal difficulty, and participant self-efficacy also warrant further exploration and may be important variables to consider when stratifying subjects (pre-randomisation) in future lifestyle intervention trials [28,29]. The GAS process in the current study provides a nuanced and bespoke method of quantifying, measuring, and comparing an individual’s lifestyle goals, ensuring that outcomes and outcome measures are patient-centric and patient-important [30]. Ultimately, more adequately powered studies are required to assess the utility of the GAS as a primary outcome measure in lifestyle trials.

As reported in the U-DECIDE study primary outcomes publication [17], though 94% of the intervention participants had a higher frequency of outpatient dietetic and exercise specialist contact than the comparator group, only 42% of the intervention participants attended ≥ 80% of the scheduled video consultation dietetic and/or exercise sessions. Insufficient exposure to the digital health exercise and diet service may therefore also explain why there was no difference in goal outcomes between the intervention and comparator groups. Furthermore, goal attainment was a secondary outcome in this study and was therefore not the basis for the sample size calculations. Low exposure to the intervention and underpowering therefore limits the certainty of these findings.

Participants reported similar improvements in dietary goal achievement in both intervention and comparator groups. However, participants who reported higher nutrition self-efficacy at baseline were more likely to report improvements on their dietary goals at the end of the study. Similar findings have been noted by others who have studied nutrition self-efficacy [31,32]. The results in our study may be explained given that dietary goals had a lower average degree of difficulty than fitness or weight loss goals. Contrarily, the current study found that physical exercise self-efficacy did not correlate with improvements in fitness goals. Interestingly, exercise self-efficacy has shown mixed results as a predictor of improved exercise outcomes in other lifestyle modification trials [33,34]. At least in people with complex medical conditions, exercise self-efficacy may be less reliable than nutrition self-efficacy in predicting relevant outcomes.

This study, which was completed during the COVID-19 pandemic, demonstrated the use of goal setting and the GAS as an outcome measure in an RCT assessing lifestyle intervention. However, the pandemic did necessitate the early cessation of recruitment, ultimately leading to underpowering for secondary outcomes including the GAS. For this analysis to have the statistical power to adequately compare GAS scores between groups (powered at 80% and at 5% statistical significance), 382 participants (191 per group) would have been required. Furthermore, the pandemic uniquely and independently influenced exercise and dietary habits for people, including participants in this study [35,36,37,38]. The results may thus be different in the non-pandemic setting. Similarly, participants were recruited from a single centre, thus potentially limiting the generalisability of these findings outside of a tertiary or transplant hospital setting. Relative to the comparator group, the intervention group did not demonstrate any statistically significant differences in goal outcome or GAS scores. However, this may relate to the significant between-group differences in goal categories at baseline. There was a significantly higher number of dietary goals in the comparator group and a significantly higher number of fitness goals in the intervention group. Given that dietary goals had a lower degree of difficulty compared to fitness goals, one might have expected the comparator group to significantly outperform the intervention group. The lack of difference in goal outcome or GAS scores between groups, despite the intervention participants having ‘tougher’ goals, may therefore suggest that digital health diet and exercise services did potentially have a meaningful impact on goal attainment. Comparator and intervention groups may have been better balanced at baseline with a greater number of participants in the study. Potentially due to the novelty of the study, it was challenging to compare and contrast the main GAS and goal attainment findings with other similar studies. Other limitations of the study included reliance on participant recall when reviewing dietary goals at the follow-up visit. In contrast, participants had access to objective data during the study (e.g., step counts, heart rate monitor, and scales), which may have impacted their reporting of fitness and weight loss goal achievement. As with other studies that rely on dietary recall, the dietary goal data were likely influenced by recall bias and social desirability bias [39].

## 5. Conclusions

The novelty and feasibility of goal setting and goal attainment were demonstrated in this RCT of lifestyle interventions in people with kidney and liver disease at increased cardiometabolic risk. Common participant goals were related to weight loss (46%), fitness (29%), and diet (15%). Though exposure to the intervention did not significantly improve goal attainment, 62% of participants reported improvements in their lifestyle goals. There were more significant improvements in dietary goals, compared with fitness or weight loss goals, and participants who reported higher nutrition self-efficacy at baseline were more likely to improve on their dietary goals. Fitness and weight loss goals were more difficult to achieve, and though this may have reflected ambitious goal setting, it may also highlight the need for broader support for people with these goals. Goal setting, participant self-efficacy, and the GAS have potentially important implications for future lifestyle modification research and clinical implementation endeavours.

## Figures and Tables

**Table 1:** Baseline demographics and medical history of participants.

Variable	Comparator	Intervention	Overall
	(n = 34)	(n = 32)	(n = 66)
**Age (mean, SD)**	48.8 (12.3)	54.0 (14.3)	51.0 (13.3)
**Male gender (n, %)**	20 (59%)	17 (53%)	37 (56%)
**Chronic disease (n, %)**			
Kidney	22 (65%)	24 (75%)	46 (70%)
Liver	12 (35%)	8 (25%)	20 (30%)
**Stage of chronic disease (n, %)**			
Kidney Disease			
CKD	14 (41%)	8 (25%)	22 (33%)
Transplant	4 (12%)	12 (38%)	16 (24%)
HD	2 (6%)	2 (6%)	4 (6%)
PD	2 (6%)	2 (6%)	4 (6%)
Liver Disease			
Transplant	9 (26%)	4 (13%)	13 (20%)
MASLD/MASH/other	3 (9%)	4 (13%)	7 (11%)
**Cause of kidney disease (n, %)**			
Glomerulonephritis	7 (21%)	9 (28%)	16 (24%)
Diabetic nephropathy	2 (6%)	5 (16%)	7 (11%)
Other/unknown	2 (6%)	5 (16%)	7 (11%)
ADPKD	5 (15%)	1 (3%)	6 (9%)
Congenital nephropathy	3 (9%)	3 (9%)	6 (9%)
Hypertension/vascular	3 (9%)	1 (3%)	4 (6%)

CKD—chronic kidney disease; HD—haemodialysis; PD—peritoneal dialysis; MASLD—metabolic dysfunction-associated steatotic liver disease; MASH—metabolic dysfunction-associated steatohepatitis; ADPKD—autosomal dominant polycystic kidney disease.

**Table 2:** Frequency of individual dietary and fitness goals.

Dietary Goals	Frequency	Fitness Goals	Frequency
Increased fruit/vegetables	4	Walking/steps	22
Reduce snacks/junk/processed food	4	Cycling	4
Increase seafood	2	Running	4
Reduce carbohydrates	2	Resistance training	3
Calorie target	1		
Reduce sweetened beverages	1		
Reduce salt in diet	1		
Increase hydration	1		
Reduce days stressing about food decisions	1		

**Table 3:** Comparison of goal attainment scale scores between groups.

Variable	Comparator	Intervention	Overall	*p*-Value
	(n = 34)	(n = 32)	(n = 66)	
**Baseline GAS Score**				0.535
Median (IQR)	30 (26, 31)	30 (26, 38)	30 (26, 32)	
Range	25, 40	25, 44	25, 44	
**Outcome GAS Score**				0.714
Median (IQR)	37 (30, 45)	38 (30, 42)	38 (30, 44)	
Range	25, 75	25, 60	25, 75	
**Change in GAS Score**				0.578
Median (IQR)	7 (0, 18)	6 (0, 10)	6 (0, 12)	
Range	−12, 50	−10, 34	−12, 50	

GAS—goal attainment scale; IQR—interquartile range.

**Table 4:** Comparison of baseline self-efficacy scores between groups.

	Comparator	Intervention	Overall	*p*-Value
	(n = 34)	(n = 32)	(n = 66)	
**Nutrition Self-efficacy Score**				0.31
Median (IQR)	17 (15, 19)	15 (14, 19)	17 (14, 19)	
Range	10, 20	7, 20	7, 20	
**Physical Exercise Self-efficacy Score**				0.28
Median (IQR)	14 (10, 15)	14 (12, 17)	14 (11, 16)	
Range	5, 20	5, 20	5, 20	
**Composite Self-efficacy Score**				0.85
Median (IQR)	31 (26, 34)	31 (25, 34)	31 (25, 34)	
Range	17, 40	12, 40	12, 40	

IQR—interquartile range.

**Table 5:** Correlation analysis of Physical Self-efficacy, Nutrition Self-efficacy, and Change in GAS.

Self-Efficacy Score	Change in GAS	
**Physical Exercise Self-efficacy**	Spearman’s rho	−0.12
	*p*-value	0.50
**Nutrition Self-efficacy**	Spearman’s rho	0.51
	*p*-value	0.04

## Data Availability

The original contributions presented in the study are included in the article, further inquiries can be directed to the corresponding author.
